# Defining Activity‐Based Subgroups in Multiple Sclerosis: A Review and Framework Proposal

**DOI:** 10.1111/ene.70663

**Published:** 2026-06-10

**Authors:** Catalina Lopez Manzano, Claire M. Rice, Emma Tallantyre, Chris Cooper, Hanyu Wang, Eve Tomlinson, Ayman Sadek, Ananya Rao‐Middleton, Howard Thom, Penny Whiting

**Affiliations:** ^1^ Bristol Technology Assessment Group (TAG), Population Health Sciences, Bristol Medical School University of Bristol Bristol UK; ^2^ School of Psychology and Neuroscience University of Bristol Bristol UK; ^3^ Department of Neurology North Bristol NHS Trust Bristol UK; ^4^ Division of Psychological Medicine and Clinical Neurosciences, School of Medicine Cardiff University Cardiff UK; ^5^ Patient Representative Bristol UK

**Keywords:** clinical trials, evidence synthesis, multiple sclerosis, relapsing–remitting, review

## Abstract

**Background:**

Multiple sclerosis (MS) is commonly stratified into activity‐based subgroups to inform treatment decisions and health technology assessments (HTAs). However, the terminology and criteria used to define these subgroups, particularly ‘active’, ‘highly active’ and ‘rapidly evolving severe’ (RES) disease, are inconsistent across clinical trials and regulatory guidance, leading to ambiguity in practice and restricted treatment access. Our objectives were to review how activity‐based subgroups in MS are defined across NICE technology appraisals (TAs) and the randomised controlled trials (RCTs) that inform them, and to propose a preliminary framework for standardised terminology.

**Methods:**

We identified and reviewed NICE TAs of disease‐modifying therapies (DMTs) for MS and the RCTs included in those appraisals. We extracted and compared definitions used to characterise activity‐based MS subgroups, and classified them by key components such as relapse history, MRI findings and treatment status. We summarised our findings and highlighted points of consensus and controversy.

**Results:**

Definitions varied widely, especially for ‘highly active’ RRMS and active SPMS. While most TAs used consistent criteria for ‘active’ and ‘RES’ RRMS disease, definitions in RCTs were heterogeneous. We identified frequent overlaps in terminology and proposed a simplified framework comprising three distinct but overlapping subgroups for RMS: active RRMS/RRMS, highly active RRMS (HARRMS) and rapidly evolving severe RRMS (RES‐RRMS).

**Conclusions:**

Standardised definitions are urgently needed to improve evidence synthesis, inform regulatory guidance and ensure equitable treatment access. We propose a formal consensus process to consolidate this framework for use in future research, HTAs and treatment pathways.

## 1 Background

Multiple sclerosis (MS) is an immune‐mediated, inflammatory chronic disease that affects the central nervous system (CNS) [1, 2]. It is the most common cause of non‐traumatic disabling disease in young adults in the United Kingdom [3]. MS may lead to the accumulation of neurological disability, including impaired mobility and cognition, although the introduction of disease‐modifying therapies (DMTs) has altered the natural history, including a reduction in relapse number and slowing disease progression [4].

MS disease activity has historically been classified into three subgroups based on clinical patterns: relapsing–remitting MS (RRMS), primary progressive MS (PPMS) and secondary progressive MS (SPMS). These subgroups were defined by the United States Advisory Committee on Clinical Trials of New Agents in Multiple Sclerosis for the National Multiple Sclerosis Society (NMSS) in 1996 [5], and revised in 2014 [6]. An additional subgroup that includes people with RRMS and active SPMS, called relapsing multiple sclerosis (RMS), has been widely used in research and policy because of the clinical similarities between these two populations [7]. These subgroups and their definitions have traditionally been used to guide patient care, research and regulatory approval of medications [8]. However, there are now calls to move away from these clinically defined subgroups to biologically based definitions aligned to the pathophysiology of the condition and recognise that MS is a continuum of disease activity, with alternatingly dominant relapsing and progressive components [8, 9].

More recently, studies and treatment algorithms have attempted to define additional sub‐categories of MS according to the level of inflammatory disease activity [10]. Various terms have been used to describe these subgroups; however, there is no consensus on how they should be named or defined. People with RRMS will often start DMT when they are determined to have ‘active’ disease, and DMTs for SPMS are currently only approved for patients with evidence of inflammatory activity. Additionally, there is evidence that people with a more aggressive disease course of RRMS, frequently called ‘highly active’ but also named ‘aggressive MS’ or ‘malignant MS’, who present with more frequent and disability‐accruing relapses at disease onset, are at an increased risk of progression and worse long‐term outcomes. This includes people with persistent activity despite receiving treatment and people with particularly high activity levels, irrespective of treatment [10, 11], commonly known as rapidly evolving severe (RES) MS in the United Kingdom.

In the United Kingdom, the National Institute for Health and Care Excellence (NICE) assesses the clinical and cost‐effectiveness of new interventions through its Technology Appraisal (TA) Programme [12]. Companies requesting approval for their drugs provide an evidence submission, usually based on evidence from randomised controlled trials (RCTs) assessing the effectiveness of these drugs and an economic model looking at cost‐effectiveness [13]. The NHS treatment algorithm for DMT for MS incorporates recommendations from NICE TAs and is based on disease activity. For the subgroup of people with RRMS, it specifies recommended treatments for: first‐line therapy of active RRMS; RES and second‐ and third‐line therapy for people with disease activity under treatment. Similarly, the algorithm recommends treatment for people with active SPMS and includes disease activity requirements in the treatment starting criteria for PPMS. However, definitions are not consistent for these sub‐categories and are dependent on definitions used in the NICE TAs that recommend specific treatments, and these also vary. The lack of consensus on terms and definitions for different MS sub‐populations has also added uncertainty to the TA development processes [14], impacting the ability to compare treatment efficacy and introducing accessibility constraints for doctors and patients when selecting a treatment.

## 2 Methods

This study provides a methodological review of the varying definitions of activity‐based MS subpopulations used in NICE TAs and clinical trials of DMTs for MS. It is reported in accordance with the PRISMA 2020 guidelines [15], with adaptations appropriate for a methodological review rather than a systematic review of intervention effects. We include definitions from TAs and trials included in each appraisal and categorise these to show where there are similarities and differences across definitions. We also identify common components among the included definitions and propose a preliminary framework to address any inconsistencies that we identified.

### 2.1 Eligibility Criteria

NICE TAs of DMTs for the treatment of MS were eligible for inclusion. TAs with status marked as ‘Published’ and ‘In development’ were included, but those labelled as ‘Awaiting development’ or ‘Topic selection’ were excluded. We excluded withdrawn and updated TAs because they were not publicly available. We also included all RCTs used as evidence of clinical effectiveness in eligible TAs. There were no restrictions based on language or publication date. TAs or RCTs restricted to people with clinically isolated syndrome (CIS) were excluded because disease activity subgroups are not relevant for this subpopulation.

### 2.2 Data Sources

We searched the NICE website on 12 June 2025, to identify eligible TAs [16]. We identified guidance related to MS by selecting the ‘Browse guidance by topic’ option. We then selected ‘neurological conditions’ and then ‘multiple sclerosis’ and selected to view ‘Published products on this topic’. We filtered further to ‘Guidance’, ‘Published’ and ‘In development’. We identified eligible RCTs by screening the included TAs to identify RCTs included in these appraisals.

### 2.3 Data Extraction and Synthesis

Data extraction was performed in Microsoft Excel, with separate sheets for TAs and RCTs. We extracted the following data from each report: report type (TA or RCT), study name or TA identifier, DMTs evaluated and population included (RRMS, RMS, CIS, SPMS, PPMS or any combination). We extracted definitions used to describe each of the following activity‐based MS sub‐populations: active RRMS/RMS (analysed as a single group because of the significant overlap between its populations), active SPMS, active PPMS, highly active RRMS disease and RES‐MS. Relevant data were extracted from the scope or background section of NICE TAs, as well as from inclusion criteria or sub‐populations defined in RCTs. Where highly active disease was categorised further as disease activity under DMT treatment and/or disease activity irrespective of previous treatment, we also extracted definitions used for these categories and different populations. We copied definitions exactly as reported in the original reports.

We reviewed the definitions extracted from the original study reports. Then we classified these according to the components of the definitions: number of relapses, periods for the number of relapses, any additional conditions not dependent on the number of relapses, whether evidence of magnetic resonance imaging (MRI) activity was required, description of MRI findings in T1 and T2 weighted images and any criteria related to previous treatment. We then collated the number of TAs and RCTs that fulfilled each of the possible combinations of criteria.

## 3 Results

We identified 21 TAs that assessed DMTs for the treatment of MS. One has been withdrawn, and two have been updated and replaced by other TAs and were excluded from this review. Eighteen TAs were included: 16 were published, and two were ‘in development’. From these 18 TAs, 16 assessed DMTs on ‘RRMS/RMS’: one targeted people with any form of MS [17], four [18, 19, 20, 21, 22, 23, 24, 25] focused on ‘RMS’, seven [22, 23, 24, 25, 26, 27, 28] on ‘RRMS’, four [29, 30, 31, 32] on ‘highly active RRMS’, one [33] assessed people with PPMS and one [34] assessed people with SPMS. The TA which evaluated people with MS in general also presented results for the SPMS group. Table S1 provides an overview of TAs included in this review.

The 18 identified TAs included 72 RCTs. Five of these RCTs were excluded because they were restricted to CIS populations. Sixty‐seven RCTs were thus included in the review, with 62 enrolling RMS participants (18 included people with RMS, 43 focused on RRMS and 1 on MS in general), 4 RCTs included people with SPMS and 1 included people with PPMS. A comprehensive list of all the TAs and RCTs included in this review, as well as the data extracted from each, is provided in Tables S2 and S3.

## 4 Active Disease

### 4.1 Active RRMS/RMS Disease

Twelve out of 16 TAs (75%; assessing 15 DMTs) and 49 out of 62 RCTs (79%; assessing 17 DMTs) provided a definition for ‘active RRMS/RMS disease’. Ten TAs specified that the population included in the appraisal was those with active RRMS (the four TAs that included people with RMS ultimately gave recommendations exclusively for people with RRMS due to a lack of evidence in the active SPMS population). Two [31, 32] defined the population of interest as those with highly active RRMS, but also presented a definition for ‘active’ disease. Thirty‐six RCTs included participants with RRMS, and 13 included participants with RMS. Two TAs and eight RCTs included people with active disease, but did not report how this population was defined, and two TAs and five RCTs did not include people with active RRMS. All TAs used the name ‘active disease’ to describe this sub‐population. Most RCTs did not specify a name for this population but used a definition of active disease as inclusion criteria for those eligible for specific DMT treatment. Those who named the population also used the term ‘active disease’.

The TAs and RCTs used broadly similar definitions of ‘active disease’. The most commonly used definition of ‘active disease’ was ‘at least two relapses in the past two years’. This definition was used on its own in 10 (83.3%) of the TAs and 9 (18.4%) of the RCTs, and as a component of the definition in 11 (91.7%) of the TAs and 25 (51.0%) of the RCTs. The remaining TA defined active disease as ‘at least 1 relapse in the past year’. Definitions used in the RCTs were more variable, but all were based on relapses in the past 1, 2 or 3 years, with some including requirements for MRI activity. Tables 1 and 2 provide an overview of the different definitions of active disease used in the TAs and RCTs.

**TABLE 1:** Overview of definitions of ‘active’ used in the included TAs for each MS population.

Description	Additional MRI activity required	RRMS/RMS (*n* = 12)	SPMS (*n* = 2)	PPMS (*n* = 1)
≥ 2 relapses in 2 years	x	10	0	0
≥ 1 relapse in 1 year	x	1	0	0
≥ 1 relapse in 2 years plus MRI activity or ≥ 2 relapses in 2 years	✓—≥ 1 T1 Gd+ lesion at screening	1	0	0
x	✓—≥ 1 T1 Gd+ lesion at screening or baseline or new T2 lesions between screening and baseline	0	0	1
≥ 1 relapse in 2 years and/or MRI activity	✓—≥ 1 T1 Gd+ lesion at baseline	0	1	0
Continuing relapses	x	0	1	0

Abbreviations: Gd+, gadolinium‐enhancing; MRI, magnetic resonance imaging; MS, multiple sclerosis; PPMS, primary progressive multiple sclerosis; RMS, relapsing multiple sclerosis; RRMS, relapsing–remitting multiple sclerosis; SPMS, secondary progressive multiple sclerosis; TAs, technology assessments.

**TABLE 2:** Overview of definitions of ‘active’ used in the included RCTs for each MS population.

Description	Additional MRI activity required	RRMS/RMS (*n* = 49)	SPMS (*n* = 4)	PPMS (*n* = 1)
≥ 1 relapse in 1 year	x	6	0	0
≥ 1 relapse in 1 year or ≥ 2 relapses in 2 years	x	7	0	0
≥ 1 in previous year, 2 in previous 2 years, or 1 in previous 1–2 years plus MRI activity	≥ 1 T1 Gd+ lesion within 1 year before randomisation	1	0	0
≥ 1 relapse in 1 year or ≥ 2 relapses in 2 years or MRI activity	≥ 1 T1 Gd+ lesion within 6 months to 1 year before randomisation	5	0	0
	≥ 1 T1 Gd+ lesion at screening	1	0	0
≥ 1 relapse in 1 year or MRI activity	≥ 1 T1 Gd+ lesion within 6 weeks before randomisation	2	0	0
≥ 1 relapse in 1 year plus MRI activity	≥ 1 T1 Gd+ lesion at screening	1	0	0
	≥ 1 T1 Gd+ lesion or ≥ 9 T2 lesions at screening	1	0	0
≥ 1 relapse in 1 year plus MRI activity or MRI activity in the past 6 weeks	Lesions consistent with multiple sclerosis, or ≥ 1 T1 Gd+ lesion or lesions consistent with MS within 6 weeks of randomisation	1	0	0
≥ 1 relapse in 2 years plus MRI activity	≥ 1 T1 Gd+ lesion at screening	1	2	0
	≥ 3 Lesions consistent with MS at screening	1	0	0
≥ 1 relapse in 6 months plus MRI activity	≥ 1 T1 Gd+ lesion within 6 months before randomisation	1	0	0
≥ 2 relapses in 2 years	x	9	0	0
≥ 2 relapses in 2 years, with ≥ 1 relapse in previous year	x	2	0	0
≥ 2 relapses in 3 years	x	2	0	0
≥ 2 relapses in 3 years, with ≥ 1 relapse in previous year	x	3	0	0
≥ 1 relapse in 1 year or ≥ 1 relapse in 2 years plus MRI activity	≥ 1 T1 Gd+ lesion within 1 year before randomisation	3	0	0
≥ 1 relapse in 1 year plus MRI activity or ≥ 2 relapses in 2 years	≥ 1 T1 Gd+ lesion at screening	1	0	0
≥ 1 relapse in 18 months	x	1	0	0
1 relapse in 6 months plus disease progression	x	0	1	0
≥ 2 relapses in 2 years or disease progression	x	0	1	0
≥ 1 T1 Gd+ lesion or new T2 lesions between screening and baseline		0	0	1

Abbreviations: Gd+, gadolinium enhancing; MRI, magnetic resonance imaging; MS, multiple sclerosis; PPMS, primary progressive multiple sclerosis; RCTs, randomised controlled trials; RMS, relapsing multiple sclerosis; RRMS, relapsing–remitting multiple sclerosis; SPMS, secondary progressive multiple sclerosis.

### 4.2 Active SPMS

Two TAs and four RCTs defined ‘active disease’ in the SPMS subgroup. The definitions observed a similar format to those in the RRMS/RMS population although two RCTs [35, 36] included disease progression as an additional component. All the definitions include an optional or mandatory requirement of a minimal number of relapses in a set period of time, with some evidence of MRI activity as an additional or alternative qualifying criterion. Two RCTs include evidence of disease progression in a defined time period. There was no consensus on any of these components. It is important to note that the 13 RCTs which included participants with RMS contained a small proportion of participants with ‘active SPMS’, for whom the ‘active RRMS/RMS’ definition was applied at enrolment. Further details of the included definitions are shown in Tables 1 and 2.

### 4.3 Active PPMS

Only one TA [33] and one RCT [37] include an ‘active’ definition for the PPMS subgroup, and both used the same MRI‐activity‐dependant definition: participants with ≥ 1 T1 Gd+ lesion at screening or baseline or new T2 lesions between screening and baseline (See Tables 1 and 2).

## 5 Highly Active Disease

Fifteen TAs (94%) and 13 RCTs (21%) included a definition for a population considered to have increased disease activity or highly active RRMS disease. No TAs or RCTs conducted exclusively in SPMS or PPMS populations defined highly active disease because this concept is not clinically relevant to these phenotypes.

### 5.1 Components of the Definitions of Highly Active Disease

Definitions of highly active disease varied across studies. We summarised them in Table 3. Reports generally used a combination of the following categories, with participants falling into any of these categories considered to have highly active disease:

**TABLE 3:** Overview of ‘highly active’ disease definitions used in the included TAs and RCTs.

1a. Unchanged/increased relapse rate or ongoing severe relapses compared with the previous year, despite treatment	1b. ≥ 1 relapse in the previous year while on treatment (Additional MRI activity required)	2. ≥ 2 relapses in a year, regardless of treatment, plus MRI activity (often called RES)	Other	TAs (*n* = 15)	RCTs (*n* = 13)
✓—Any DMT	x	x	x	4	0
✓—IFN‐β	x	x	x	2	0
✓—Any DMT	✓—Any DMT (≥ 1 T1 Gd+ lesion or ≥ 9 T2 lesions)	x	x	1	2
✓—IFN‐β	✓—IFN‐β (≥ 1 T1 Gd+ lesion or ≥ 9 T2 lesions)	✓—≥ 1 T1 Gd+ lesion or significant increase in T2 lesion load	x	1	0
✓—Any DMT	✓—Any DMT (≥ 1 T1 Gd+ lesion or ≥ 9 T2 lesions)	✓—≥ 1 T1 Gd+ lesion or significant increase in T2 lesion load	x	1	0
✓—Any DMT	✓—Any DMT (≥ 1 T1 Gd+ lesion or ≥ 9 T2 lesions)	✓—≥ 1 T1 Gd+ lesion	x	0	1
✓—Any DMT	✓—Any DMT (≥ 1 T1 Gd+ lesion or T2 lesion volume > 0.5 mL)	✓—≥ 1 T1 Gd+ lesion	x	0	1
x	✓—IFN‐β (≥ 1 T1 Gd+ lesion or ≥ 9 T2 lesions)	x	x	1	2
x	✓—IFN‐β (≥ 1 T1 Gd+ lesion or ≥ 9 T2 lesions)	x	x	2	0
x	✓—IFN‐β (No MRI evidence required)	x	x	0	1
x	x	✓—≥ 1 T1 Gd+ lesion	x	0	3
x	✓—Any DMT (≥ 1 T1 Gd+ lesion or ≥ 9 T2 lesions)	✓—≥ 1 T1 Gd+ lesion	x	1	0
x	✓—Any DMT (≥ 1 T1 Gd+ lesion or ≥ 9 T2 lesions)	✓	x	1	1
x	x	x	✓	1	2

Abbreviations: Gd+, gadolinium‐enhancing; MRI, magnetic resonance imaging; MS, multiple sclerosis; RCTs, randomised controlled trials; RES, rapidly evolving severe; TAs, technology assessments.

Category 1: Disease activity under treatment:

Category 1a: Unchanged or increased relapse rate or ongoing severe relapses compared with the previous year, despite treatment.

Category 1b: At least one relapse in the past year while on treatment, with variable requirement for MRI evidence of activity.

Category 2: Disease activity regardless of treatment, *often referred to* as ‘rapidly evolving severe’ *MS*.

Definitions from 11/15 TAs and 7/13 RCTs consisted of only one of these components; definitions in others were composed of two or more of these components. In 11 TAs and seven RCTs, people required breakthrough activity while on DMT treatment (Category 1) to be classified as having ‘highly active’ disease. Definitions from four TAs and three RCTs allowed both Category 1 and people with increased activity, irrespective of any previous treatment (Category 2). Definitions for the remaining three RCTs only included participants with increased activity regardless of previous treatment (Category 2). When DMT treatment was a requirement, some definitions specified a particular treatment: five TAs and one RCT only considered prior interferon beta treatment, two TAs and two RCTs considered either prior interferon beta (INF‐β) or glatiramer acetate; the other reports included treatment with any DMT. Where evidence of MRI activity was required, this was usually a combination of at least one gadolinium‐enhancing lesion or at least 9 T2 lesions or a ‘significant’ increase in T2 lesion load.

One TA [18] and two RCTs used definitions that were not consistent with the categories outlined above. The TA simply defined a population with highly active disease as ‘patients who discontinued their last DMT due to lack of efficacy’. One RCT [38] defined highly active disease as ‘≥ 2 relapses in the year before study entry and ≥ 1 gadolinium‐enhancing lesion in T1 weighted MRI (T1 Gd+ lesion) at study entry while on treatment with INF‐β or glatiramer acetate’ and one [39] defined it as ‘Patients with at least two attacks in the previous 2 years with at least one in the previous year; at least one relapse while on interferon beta or glatiramer after at least 6 months of treatment’.

### 5.2 Terminology for Highly Active Disease

There was a lack of consistency in terminology used to describe those with highly active disease. The different names used for this population, along with a summary of how these relate to the various categories used to define highly active disease, are shown in Table 4. Some reports categorised highly active disease further based on whether it was or was not dependent on treatment; these therefore have multiple different subgroups, based on high disease activity, listed in Table 3.

**TABLE 4:** Overview of names and definitions of highly active disease used in the included TAs and RCTs.

Terminology	Definition category				TAs	RCTs
	1a	1b	2	Other		
Highly active	✓	x	x	x	5	0
	x	✓	✓	x	2	0
	x	✓	x	x	2	2
	✓	✓	x	x	1	2
	✓	✓	✓	x	1	2
	x	x	✓	x	0	2
	x	x	x	✓	0	1
	x	x	x	✓	1	0
High disease activity (HDA)	x	✓	✓	x	1	1
Highly active despite previous treatment	x	✓	x	x	3	0
High disease activity despite treatment	x	✓	x	x	1	1
Disease activity on treatment (DAT)	x	✓	x	x	0	1
Sub‐optimally treated	x	✓	x	x	3	0
Active relapsing disease despite interferon beta treatment	x	✓	x	x	0	1
High relapse activity (HRA)	x	x	✓	x	0	1
Treatment naïve, highly active	x	x	✓	x	0	1
Rapidly evolving severe (RES)	x	x	✓	x	15	3
Not specified	x	x	x	✓	0	1
	✓	✓	x	x	0	1
	x	x	✓	x	0	1

*Note:* 1a: Unchanged or increased relapse rate or ongoing severe relapses compared with the previous year, despite treatment.

1b: At least one relapse in the past year while on treatment, with variable requirement for MRI evidence of activity.

2: Disease activity regardless of treatment.

Abbreviations: RCTs, randomised controlled trials; TAs, technology assessments.

The most frequently used name was ‘highly active disease’—this was used in 12 TAs and 9 RCTs. However, within these reports, there were at least eight different ways of defining what they considered to be a highly active disease. Some reports used different names to represent effectively the same population. For example, studies that defined highly active disease as category 1b (‘At least one relapse in the past year while on treatment, sometimes with additional MRI evidence of activity’) used the following names for this population: ‘highly active’, ‘highly active despite previous treatment’, ‘high disease activity despite treatment’, ‘disease activity on treatment (DAT)’, ‘sub optimally treated’ and ‘active relapsing disease despite INF‐β treatment’.

### 5.3 RES Disease

Fifteen out of 18 TAs and 8 out of 67 RCTs described a subgroup with increased activity, irrespective of previous DMT treatment, often labelled as ‘Rapidly evolving severe disease (RES)’ in the RRMS subgroup. One TA that focused on participants with SPMS [34] described a subgroup called ‘Rapidly evolving severe’, but it was not disease activity‐based and was defined as participants with an ‘EDSS change ≥ 1.5 in the 2 years prior to or at study start’. Furthermore, the corresponding RCT [40] named this subgroup ‘rapid progression’ instead.

Four out of 15 TAs and six out of eight RCTs considered RES to be a subgroup of the highly active disease population. These, therefore, included people with RES in their definitions of highly active disease and are listed in Tables 3 and 4 as ‘Category 2’ (defined above). Eleven TAs and two RCTs considered this group to be independent of the ‘highly active’ population; therefore, they are not considered in the discussion of this population.

All 15 TAs used the label ‘Rapidly evolving severe disease (RES)’, but only three of the eight RCTs used this term. Two RCTs [41] described this subgroup as having ‘highly active’ disease, one [38] as ‘treatment‐naïve highly active’, one [42] ‘high relapse activity (HRA)’ and one [43] did not give this population a specific name.

The definition of RES was broadly similar across reports, as showcased in Table 5. Most reports (14 TAs and 7 RCTs) defined rapidly evolving disease as at least two relapses in 1 year, combined with evidence of MRI activity. One TA [26] defined RES as at least one relapse in the past year combined with evidence of MRI activity, and one RCT defined RES as at least 2 relapses in the past year, irrespective of MRI activity. Where evidence of MRI activity was required, all reports defined this as the presence of at least one gadolinium‐enhancing lesion on T1 images; some also considered a significant increase in T2 lesion load compared with a previous MRI as evidence of MRI activity.

**TABLE 5:** Overview of RES definitions used in TAs and RCTs.

Number of relapses in the past year	MRI activity—T1	MRI activity—T2	Is RES considered a subgroup of highly active?	TAs (*n* = 15)	RCTs (*n* = 8)
≥ 2	≥ 1 T1 Gd+ lesion or	Significant increase in T2 lesion load	No	8	2
			Yes	2	0
≥ 2	≥ 1 T1 Gd+ lesion	x	No	2	0
			Yes	1	5
≥ 2	Baseline evidence of disease	x	No	1	0
≥ 2	x	x	Yes	0	1
≥ 1	≥ 1 T1 Gd+ lesion	Significant increase in T2 lesion load	No	1	0

Abbreviations: Gd+, gadolinium‐enhancing; MRI, magnetic resonance imaging; RCTs, randomised controlled trials; RES, rapidly evolving severe; TAs, technology assessments.

## 6 Discussion

This review highlights substantial inconsistencies in how activity‐based subgroups of MS are defined and labelled across NICE TAs and the RCTs that inform them. We identified 18 TAs and 67 RCTs that included MS subgroups based on disease activity. While definitions of ‘active’ RRMS/RMS and ‘rapidly evolving severe’ (RES) disease show some alignment, particularly across TAs, the definitions used in RCTs are more variable. In contrast, terminology and criteria for ‘highly active’ disease vary markedly across both sources. Some studies apply the same label to different subgroups, while others use different labels to describe similar populations. Our findings also reveal that terms such as ‘active MS’, ‘highly active MS’ and ‘RES’ are often used interchangeably, even when referring to only partially overlapping or entirely distinct populations. There is not enough data to draw conclusions for the SPMS and PPMS subgroups, but the very limited data available already showcase that the lack of consistency extends through the whole range of clinical presentations of MS.

To our knowledge, this is the first methodological review of activity‐based MS subgroup definitions used in NICE appraisals. Its strengths include a transparent methodology and the development of a preliminary framework based on areas of historical consensus, intended to inform future policy and clinical practice. Limitations include the exclusion of real‐world evidence and the potential omission of newer trials not included in older appraisals.

Inconsistencies in terminology and definitions in RCTs complicate the interpretation of trial evidence, introduce heterogeneity into comparative analyses, and create ambiguity for clinical decision‐making and policy development. In practice, treatment algorithms and population definitions used in TAs are often based on eligibility criteria and subgroup definitions used in pivotal RCTs. As a result, the same inconsistencies and limitations embedded in population definitions used in RCTs are carried through into clinical guidance and reimbursement decisions. This linkage can restrict treatment access, particularly when subgroups defined for research purposes are applied rigidly in real‐world settings. Despite calls to move towards biologically or mechanism‐driven classification systems, activity‐based subgroups are likely to remain relevant in both research and regulatory contexts. Clinical trials require clearly defined entry criteria, and retrospective harmonisation is limited by trial design and historical decisions. Previous efforts to standardise definitions across the MS treatment landscape have largely been unsuccessful, but there is a clear need for these [8, 44].

If the use of subgroups of MS based on disease activity is to continue, then there is an urgent need to standardise definitions and terminology so that these are used consistently in future TAs and primary studies. While terms and definitions vary between TAs and RCTs, we have also identified points of consensus that we have used to propose a preliminary framework. This is a pragmatic solution that aims to clarify existing terminology and support future research, guideline development and treatment access pathways. This framework draws on areas of definitional convergence and suggests standardised subgroups to reduce ambiguity and better align trial populations with treatment guidance.

**FIGURE 1:**
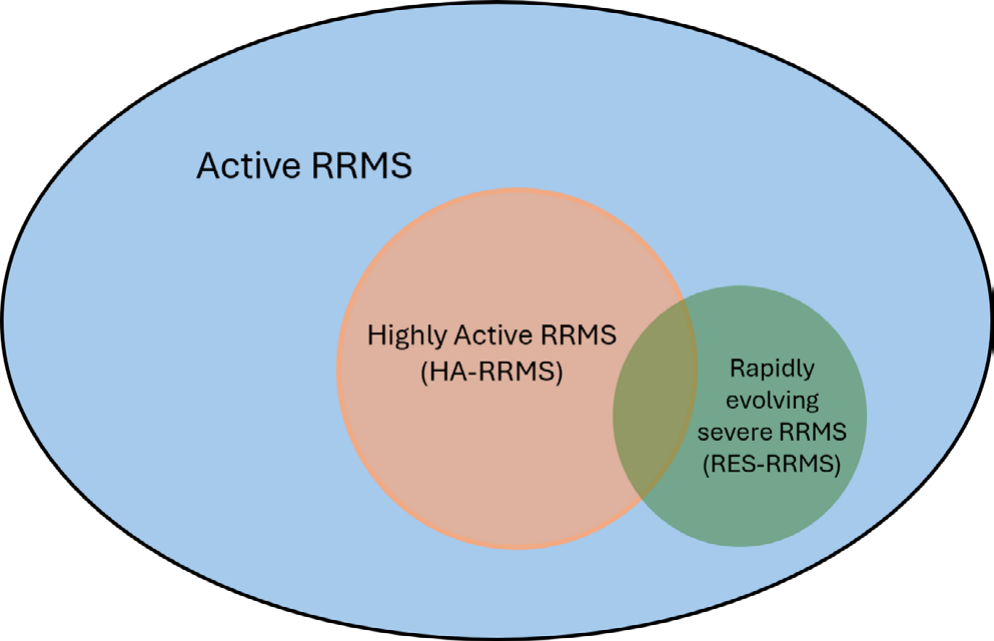
Overview of proposed categories of RRMS showing how these relate to each other.

Our preliminary framework proposes three overlapping but distinct subgroups for RMS/RRMS: active RMS/RRMS, highly active RRMS (HARRMS) and rapidly evolving severe RRMS (RES‐RRMS) (Figure 1). These categories reflect the most frequently used definitions in current appraisals and trials, though their practical application requires further refinement. To support consistency and transparency, the proposed definitions are based on the most commonly used components identified in our review. Proposed definitions are detailed in Table 6.

**TABLE 6:** Preliminary framework proposal.

Name	Definition
Active RRMS	People with ≥ 2 relapses in 2 years or ≥ 1 relapse in 1 year and/or evidence of MRI activity in the past year.
Highly active RRMS (HARRMS)	People with an unchanged or increased relapse rate, or ongoing severe relapses, compared with the previous year, despite DMT treatment; or patients who had at least one relapse and had evidence of MRI activity in the previous year while on treatment. *Note:* This should only include those with disease activity under treatment
Rapidly evolving severe RRMS (RES‐RRMS)	People with ≥ 2 relapses in a 12‐month period, regardless of treatment.

Abbreviations: DMT, disease‐modifying therapies; RRMS, relapsing–remitting multiple sclerosis.

### 6.1 Active RMS/RRMS

There is broad consensus that this subgroup should include people with MS who have experienced at least two relapses within two years. However, individuals with at least one relapse in one year and/or evidence of MRI activity during that period should also be included to ensure timely access to treatment. Areas requiring further discussion include whether these criteria must be met within a fixed timeframe prior to assessment, or at any point in the patient's history, and what should constitute MRI activity, potentially including the presence of at least one T1 gadolinium‐enhancing lesion and/or nine or more T2 lesions.

### 6.2 Active SPMS

There is very limited data available, but it already shows significant heterogeneity without enough common points to propose a preliminary definition. It is possible, however, to identify specific topics that need to be included in further discussions: the number of relapses required in a set time period, the qualifying MRI activity evidence and whether this is a mandatory or optional requirement. The inclusion of disease progression in these definitions needs to be discussed as well.

### 6.3 Active PPMS

We identified one TA with one supporting trial in this population, and both used the same definition. Some discussion is needed to assess the suitability of this definition and whether any additional components should be included.

### 6.4 HARRMS

There is a strong level of agreement on including people with an unchanged or increased relapse rate, or ongoing severe relapses, compared with the previous year, despite DMT treatment in this subgroup, as well as including people who had at least one relapse and had evidence of MRI activity in the previous year while on treatment. This subgroup should include only those with breakthrough disease activity under DMTs and should be considered distinct, although sometimes overlapping, from the RES‐RRMS group.

Further consensus is needed on whether people with MRI activity but no clinical relapses while receiving DMTs should be included, and what qualifies as meaningful MRI activity. As with the active RRMS definition, this might include people with at least one T1 gadolinium‐enhancing lesion and/or nine or more T2 lesions. Additionally, we suggest that a minimum duration on adequate treatment should be required for inclusion in this subgroup, an area that also warrants further discussion.

### 6.5 RES‐RRMS

It is widely accepted that this subgroup should include people with at least two relapses in 12 months, regardless of treatment. Additional consensus is necessary to determine whether the qualifying period should be any 12‐month period or the previous 12 months, and if there should be additional requirements for MRI activity. There is also a need for consensus regarding what would be considered evidence of MRI activity, including the timeframe of the MRI (at screening, within 6 weeks, 6 months or 1 year) and qualifying MRI parameters (e.g., ≥ 1 T1 Gd+ lesions, significant increase in T2 lesion load and/or ≥ 9 T2 lesions).

We acknowledge that these definitions are not exhaustive and that areas of uncertainty remain. To address these challenges, we propose initiating a formal consensus process involving clinicians, trialists, commissioners, patients and regulators to refine and operationalise the definitions. A key decision moving forward is whether to retrospectively harmonise existing definitions or to instead draw a line under current inconsistencies and focus on developing a forward‐looking system. We favour the latter: a proactive approach that enables better trial design, more robust evidence synthesis and more pragmatic treatment decisions in real‐world settings. Achieving this will require coordinated efforts from researchers, regulators, clinicians and patients—especially those involved in clinical trial design.

Looking ahead, it is essential to apply these lessons to future developments. As treatments for progressive MS (e.g., siponimod, ocrelizumab and tolebrutinib) become more widely available, consistent and agreed‐upon criteria will be critical to avoid repeating the definitional inconsistencies observed in RRMS subgroups. Proactively establishing clear standards for emerging populations now may help prevent similar difficulties in the future.

## Author Contributions

**Ananya Rao‐Middleton:** writing – review and editing. **Chris Cooper:** conceptualization, writing – review and editing, methodology. **Claire M. Rice:** conceptualization, methodology, writing – review and editing. **Catalina Lopez Manzano:** conceptualization, data curation, formal analysis, investigation, methodology, project administration, writing – original draft, writing – review and editing, visualization, validation. **Eve Tomlinson:** investigation, writing – review and editing. **Hanyu Wang:** investigation, writing – review and editing. **Penny Whiting:** conceptualization, data curation, formal analysis, funding acquisition, investigation, methodology, project administration, supervision, validation, visualization, writing – review and editing. **Ayman Sadek:** investigation, writing – review and editing. **Howard Thom:** supervision, writing – review and editing. **Emma Tallantyre:** conceptualization, methodology, writing – review and editing.

## Funding

This review builds upon work conducted in a Multiple Technology appraisal by Bristol Technology Appraisal Group, commissioned by the NIHR Evidence Synthesis Programme as project number NIHR 165943.

## Conflicts of Interest

The authors declare no conflicts of interest.

## Supporting information


**Table S1:** Overview of included NICE technology appraisals.
**Table S2:** Definitions of activity‐based subgroups in technology appraisals evaluating DMTs for relapsing MS.
**Table S3:** Definitions of activity‐based subgroups in RCTs evaluating DMTs for relapsing MS.

## Data Availability

The data that support the findings of this study are available from the corresponding author upon reasonable request.
