# Supplementary material for: Defining Activity‐Based Subgroups in Multiple Sclerosis: A Review and Framework Proposal
Source: Eur J Neurol. 2026 Jun 10;33(6):e70663. doi: 10.1111/ene.70663 (PMC13251442; doi:10.1111/ene.70663)
Supplement: Supplementary file 1 — Table S1: Overview of included NICE technology appraisals. Table S2: Definitions of activity‐based subgroups in technology appraisals evaluating DMTs for relapsing MS. Table S3: Definitions of activity‐based subgroups in RCTs evaluating DMTs for relapsing MS. [file ENE-33-e70663-s001.docx]

Table S1 Overview of included NICE technology appraisals.

| **TA** | **Status** | **Interventions** | **Population** |
| --- | --- | --- | --- |
| 127^1^ | Published | Natalizumab | Highly active relapsing remitting MS |
| 254^2^ | Published | Fingolimod | highly active relapsing–remitting MS |
| 303^3^ | Published | Teriflunomide | Relapsing remitting MS |
| 312^4^ | Published | Alemtuzumab | Highly active relapsing remitting MS |
| 320^5^ | Published | Dimethyl fumarate | Relapsing remitting MS |
| 527^6^ | Published | INF-β and glatiramer acetate | Multiple sclerosis |
| 533^7^ | Published | Ocrelizumab | Relapsing remitting MS |
| 585^8^ | Published | Ocrelizumab | Primary progressive MS |
| 616^9^ | Published | Cladribine | Relapsing remitting MS |
| 656^10^ | Published | Siponimod | Secondary progressive MS |
| 624^11^ | Published | Peginterferon beta-1a | Relapsing remitting MS |
| 699^12^ | Published | Ofatumumab | Relapsing MS |
| 706^13^ | Published | Ozanimod | Relapsing remitting MS |
| 767^14^ | Published | Ponesimod | Relapsing MS |
| 794^15^ | Published | Diroximel fumarate | Relapsing remitting MS |
| 1025^16^ | Published | Ublituximab | Relapsing MS |
| 1053^17^ | Published | Cladribine | Relapsing multiple sclerosis |
| ID6369^18^ | In development | Natalizumab | Highly active relapsing remitting MS |

Table S2 Definitions of activity-based subgroups in technology appraisals evaluating DMTs for relapsing MS

| **TA** | **Population** | **Active definition** | **HA name** | **Name for activity under treatment (Category 1, if different from HA name)** | **HA definition** | **Name for RES**  **(Category 2)** | **RES definition** | **Subgroups** | **Included RCTs** |
| --- | --- | --- | --- | --- | --- | --- | --- | --- | --- |
| 127^1^ | HARRMS | Active not in scope | Highly active | Sub optimally treated | Rapidly evolving severe subgroup defined by 2 or more disabling relapses in one year, and with 1 or more Gadolinium-enhancing lesions on brain Magnetic Resonance Image (MRI) or a significant increase in T2 lesion load as compared to a previous MRI and Sub optimally treated subgroup defined as patients who have had at least 1 relapse in the previous year while on therapy, and have at least 9 T2-hyperintense lesions in brain MRI or at least 1 Gadolinium-enhancing lesion. | Rapidly evolving severe (RES) | 2 or more disabling relapses in one year, and with 1 or more Gadolinium-enhancing lesions on brain Magnetic Resonance Image (MRI) or a significant increase in T2 lesion load as compared to a previous MRI | Sub optimally treated and RES are subgroups of highly active | AFFIRM^19^  MS 201^1^  MS 231^1^  SENTINEL^20^ |
| 254^2^ | HARRMS | Active not in scope | Highly active | High disease activity despite treatment | High disease activity despite treatment: These patients may be defined as those who have failed to respond to a full and adequate course (normally at least 1 year of treatment) of beta-interferon. Patients should have had at least 1 relapse in the previous year while on therapy and have at least 9 T2-hyperintense lesions in cranial MRI or at least 1 gadolinium-enhancing lesion. A ―non-responder‖ also could be defined as a patient with an unchanged or increased relapse rate or with ongoing severe relapses, as compared with the previous year Plus Patients with rapidly evolving, severe, relapsing-remitting MS defined by 2 or more disabling relapses in 1 year, and with 1 or more gadolinium-enhancing lesions on brain MRI or a significant increase in T2 lesion load as compared with a previous recent MRI. | Rapidly evolving severe (RES) | 2 or more disabling relapses in 1 year, and with 1 or more gadolinium-enhancing lesions on brain MRI or a significant increase in T2 lesion load as compared with a previous recent MRI. | RES and high disease activity subgroups of highly active | FREEDOMS I ^21^  FREEDOMS II ^22^  TRANSFORMS^23^  FTY720 D220^24^ |
| 303^3^ | HARRMS | >2 clinically significant relapses in the previous 2 years | Highly active | NA | Patients failing to respond to a full and adequate course of beta-interferon defined as at least 1 relapse in the last year and at least nine T2 hyper intense lesions or at least one gadolinium enhancing lesion. | Rapidly evolving severe (RES) | Two or more disabling relapses in one year and one or more gadolinium-enhancing lesions on brain magnetic resonance imaging (MRI) or a significant increase in T2 lesion load compared with a previous MRI | RES and HA independent | TEMSO^25^  TOWER^26^  TENERE^27^  HMR1726D/2001^28^ |
| 312^4^ | HARRMS | Two clinically significant relapses in the previous two years | Highly active | NA | Unchanged or increased relapse rate or ongoing severe relapses compared with the previous year despite treatment with IFN-β | Rapidly evolving severe (RES) | ≥2 relapses in the year prior to treatment and at least one gadolinium-enhancing lesion at baseline | RES and HA independent | CAMMS 223^29^  CARE-MS I^30^  CARE-MS II^31^ |
| 320^5^ | RRMS | 2 or more clinically significant relapses in the previous 2 years) | Highly active | NA | ≥ 1 relapse in the previous year and ≥ 9 T2-hyperintense lesions on MRI or ≥ 1 Gd+ lesion, patients who have received treatment with beta interferon | Rapidly evolving severe (RES) | ≥ 2 disabling relapses in 1 year, and with ≥1 Gd+ lesion on MRI or a significant increase in T2 lesion load as compared to a previous recent MRI | RES and HA independent | DEFINE^32^  CONFIRM^33^  Kappos 2008[#5887} |
| 527^6^ | MS | 2 clinically significant relapses in the previous 2 years | Highly active | NA | Multiple disabling relapses in a year, or unchanged relapse rate during first-line treatment. | Rapidly evolving severe (RES) | Two or more disabling relapses in 1 year, and one or more gadolinium enhancing lesions on brain magnetic resonance imaging (MRI) or a significant increase in T2 lesion load compared with a previous MRI. | RES and HA independent | ADVANCE^34^  BECOME^35^  BEYOND^36^ Bornstein 1987^37^  BRAVO^38^  Calabrese 2012^39^CombiRx{#1848  Confirm{1141}  Cop 1 MSSG^40^  ECGAS 2001^41^  ESG 1998^42^  Etemedifar 2006^43^  EVIDENCE^44^  GALA^45^  GATE^46^  IFNB^47^  IMPROVE 2012^48^ INCOMIN 2002^49^, Kappos 2011^50^ Knobler 1993^51^ MSCRG 1996^52^ Mokhber 2014^53^  NASG^54^  OWIMS^55^  PRISMS^56^ REFORMS^57^REGARD^58^ REMAIN^59^  SPECTRIMS^60^ |
| 533^61^ | RRMS | Two or more clinical relapses within two years | Highly active RRMS despite previous treatment | NA | Patients treated with interferons or glatiramer acetate for at least 1 year, and: Had at least one relapse in the previous year, and, had at least one T1 Gd-enhancing lesion on brain MRI at baseline, or Had at least nine T2 hyperintense lesions on brain MRI at baseline | Rapidly evolving severe (RES) | Patients had at least two relapses in the previous year, and: Had at least one T1 Gd-enhancing lesion on brain MRI at baseline, or had an increase in T2 hyperintense lesion count on brain MRI at baseline (changing from 0-5 to 6-9, >9 lesions or 6-9 lesions to >9 lesions), compared to previous MRI | RES and HA independent | OPERA I^62^ OPERA II^62^ |
| 585^8^ | PPMS | Evidence of gadolinium-enhancing T1 lesions at screening or baseline or new T2 lesions between screening and baseline | NA | NA | NA | NA | NA | NA | ORATORIO^63^ |
| 616^9^ | RRMS | Patients with RRMS and at least one relapse in the previous year | HDA high disease activity | Highly active despite treatment or Sub optimally treated | Patients with two or more relapses in the prior year whether on treatment or not, and at least one T1 Gd+ lesion and Patients with at least one relapse in the previous year while on DMT therapy, and at least one T1 Gd+ lesion or nine T2 lesions (RES is a subgroup) | Rapidly evolving severe (RES) | Patients with two or more relapses in the prior year whether on treatment or not, and at least one T1 Gd+ lesion | RES and HAD are subgroups of highly active | CLARITY^64^  ORACLE MS^65^ ONWARD^66^ |
| 624^11^ | RRMS | Not reported | Highly active | NA | Failure to respond to at least 1 year of treatment with a DMT and either, ≥ 1 relapse in the previous year with either ≥ 9 T2 lesions and/or ≥ 1 Gd+ lesion, or unchanged or increased relapse rate, or ongoing severe relapse compared with the previous year”. | Rapidly evolving severe (RES) | One or more disabling relapses in 1 year, and with ≥ 1 (Gadolinium) Gd+ lesion or a significant increase in T2 lesion load compared with a previous recent MRI | RES and HA independent | ADVANCE^34^ |
| 656^10^ | SPMS | Patients who experienced relapses in the two years prior to the study and/or who had gadolinium-enhanced T1 lesions at baseline. | NA | NA | NA | NA | NA | NA | EXPAND^67^ |
| 699^12^ | RMS | Not reported | Highly active | NA | Patients with RRMS previously treated with any DMT who discontinued their last DMT due to lack of efficacy. | Rapidly evolving severe (RES) | People with relapsing–remitting multiple sclerosis, who had had at least 2 relapses in the last year and at least one T1 gadolinium-enhancing lesion on baseline brain MRI | RES and HA independent | ASCLEPIOS I^68^  ASCLEPIOS II^68^ |
| 706^13^ | RMS | One relapse in the last 2 years and who also have radiological activity, or (2) two significant relapses in the last 2 years. | Highly active | NA | Unchanged or increased relapse rate, or ongoing severe relapses compared with the previous year despite treatment with at least one DMT | Rapidly evolving severe (RES) | Two or more disabling relapses in one year, and one or more gadolinium-enhancing (Gd-E) lesions on brain magnetic resonance imaging (MRI), or a significant increase in T2 lesion load compared with a previous MRI | RES and HA independent | RADIANCE Part A^69^ RADIANCE Part B^70^ RPC01-1001^71^  SUNBEAM^72^ |
| 767^14^ | RMS | ≤2 relapses in ≤2 years | Highly active | NA | An unchanged or increased relapse rate or ongoing severe relapses compared with the previous year despite treatment with beta interferon. | Rapidly evolving severe (RES) | ≥2 disabling relapses in 1 year and ≥1 gadolinium-enhancing (Gd+) lesions on brain MRI or a significant increase in T2 lesion load compared with a previous MRI. | RES and HA independent | OPTIMUM^73^  AC-058B201^74^ |
| 794^15^ | RRMS | 2 clinically significant relapses in the previous 2 years | NR | NR | NR | NR | NR | NR | EVOLVE-MS-2^32^ |
| 1025^16^ | RMS | At least two clinically significant relapses occur within the last 2 years | Highly active | NA | Unchanged/increased relapse rate or by ongoing severe relapses compared with the previous year, despite disease-modifying drug treatment. | Rapidly evolving severe (RES) | Defined by two or more disabling relapses in 1 year, and one or more gadolinium (Gd)-enhancing lesions on brain MRI or a significant increase in T2 lesion load compared with a previous MRI | RES and HA independent | ULTIMATE I^75^  ULTIMATE II^75^ |
| 1053[#5875} | RMS | Clinical or imaging features or 2 clinically significant relapses in the previous 2 years | Highly active | Sub optimally treated | (i) Patients with one relapse in the previous year and at least one T1 gadolinium (Gd) enhanced lesion or ≥9 T2 lesions, while on therapy with other DMTs, or (ii) patients with two or more relapses in the previous year, whether on DMT treatment or not) | Rapidly evolving severe (RES) | 2 or more relapses in the previous year and baseline MRI evidence of disease activity | Sub optimally treated and RES are subgroups of highly active | CLARITY^64^  ORACLE  MS^65^ ONWARD^66^ |
| ID6369^18^ | HARRMS | ≥Two clinically significant relapses within the last 2 years. | Highly active | NA | Unchanged or increased clinical or radiological evidence of disease activity despite treatment with at least one DMT | Rapidly evolving severe (RES) | ≥Two disabling relapses in 1 year and MRI changes (one or more gadolinium-enhancing lesions or a significant increase in T2 lesion load compared with a previous MRI) | RES and HA independent | AFFIRM^19^  ANTELOPE^76^  DELIVER^77^  NOVA^78^ REFINE^79^ |

Table S3 Definitions of activity-based subgroups in RCTs evaluating DMTs for relapsing MS

| **RCT** | **TA** | **DMT's evaluated** | **Population** | **Active definition /Inclusion criteria** | **HA name** | **Name for activity under treatment (if different from HA name)** | **HA definition** | **Name for RES** | **RES definition** | **RES subgroup of HA?** |
| --- | --- | --- | --- | --- | --- | --- | --- | --- | --- | --- |
| AC-058B201^74^ | 767 | Ponesimod | RRMS | ≥1 documented relapse(s) within the 12 months before screening; ≥2 documented relapses within the 24 months before screening or at least one T1-weighted gadolinium-enhanced (Gd+) lesion detected on brain MRI at screening. | NR | NR | NR | NR | NR | NR |
| ADVANCE^34^ | 527 and 624 | Peginterferon beta 1a | RRMS | At least two clinically documented relapses in the previous 3 years, with at least one having occurred within the past 12 months. | NR | NR | NR | NR | NR | NR |
| AFFIRM^19^ | 127 and ID6369 | Natalizumab | RRMS | At least one medically documented relapse within the 12 months before the study began. | Highly active disease | NR | ≥ 2 relapses in the year prior to study entry and ≥ 1 Gd+ lesion on T1-weighted MRI at study entry | Treatment naïve highly active | NR | Yes |
| ANTELOPE^76^ | ID6369 | Natalizumab | RRMS | 1 or more documented relapse within the previous year and either 1 or more gadolinium-enhancing T1-weighted or 9 or more T2-weighted brain lesions on MRI | NR | NR | NR | NR | NR | NR |
| ASCLEPIOS I^68^ | 699 | Ofatumumab and teriflunomide | RRMS | At least one relapse in the year before screening, at least two relapses in the 2 years before screening, or at least one lesion detected with the use of gadolinium enhancement (gadolinium-enhancing lesion) on magnetic resonance imaging (MRI) in the year before randomization | NR | NR | NR | NR | NR | NR |
| ASCLEPIOS II^68^ | 699 | Ofatumumab and teriflunomide | RRMS | At least one relapse in the year before screening, at least two relapses in the 2 years before screening, or at least one lesion detected with the use of gadolinium enhancement (gadolinium-enhancing lesion) on magnetic resonance imaging (MRI) in the year before randomization | NR | NR | NR | NR | NR | NR |
| BECOME^35^ | 527 | INF-β and glatiramer acetate | RRMS and CIS | Activity level not specified | NR | NR | NR | NR | NR | NR |
| BEYOND^36^ | 527 | INF-β 1b and glatiramer acetate | RRMS | At least one relapse in the year before entry into the study | NR | NR | NR | NR | NR | NR |
| BORNSTEIN 1987^37^ | 527 | Glatiramer acetate | RRMS | Two well demarcated and well-documented episodes of exacerbation in the two years before admission | NR | NR | NR | NR | NR | NR |
| BRAVO^38^ | 527 | Laquinimod and INF-β 1a | RRMS | ≥ 1 in previous year, 2 in previous 2 years, or 1 in previous 1–2 years and ≥ 1 GdE lesion in the previous year | NR | NR | NR | NR | NR | NR |
| CALABRESE 2012^39^ | 527 | INF-β 1a and glatiramer acetate | RRMS | Activity level not specified | NR | NR | NR | NR | NR | NR |
| CAMMS 223^29^ | 312 | Alemtuzumab | RRMS | At least two clinical episodes during the previous 2 year | NR | NR | NR | NR | NR | NR |
| CARE-MS I^30^ | 312 | Alemtuzumab | RRMS | At least two clinical episodes during the previous 2 year | NR | NR | NR | NR | NR | NR |
| CARE-MS II^31^ | 312 | Alemtuzumab | RRMS | At least two relapses in the previous 2 years and at least one in the previous year | NR | NR | Patients with at least two attacks in the previous 2 years with at least one in the previous year; at least one relapse while on interferon beta or glatiramer after at least 6 months of treatment | NR | NR | NR |
| CLARITY | 616 and 1053 | Cladribine | RRMS | At least one relapse within 12 months before study entry | High disease activity | Disease activity on treatment (DAT) | Patients with ⩾2 relapses during the year prior to study entry, whether on DMD treatment or not and patients with ⩾1 relapse and⩾1 T1 Gd+ or ⩾9 T2 lesions during the year prior to study entry while on therapy with other DMDs | High relapse activity (HRA) | High relapse activity (⩾2 relapses during the year prior to study entry, whether on DMD treatment or not | Yes |
| CombiRx^80^ | 527 | INF-β 1a and glatiramer acetate | RRMS | At least 2 exacerbations in the prior three years | NR | NR | NR | NR | NR | NR |
| CONFIRM^33^ | 320, 527 and 794 | Dimethyl fumarate and glatiramer acetate | RRMS | At least one clinically documented relapse in the previous 12 months or at least one gadolinium-enhancing lesion 0 to 6 weeks before randomization. | NR | NR | NR | NR | NR | NR |
| Cop 1 MSSG^40^ | 527 | Glatiramer acetate | RRMS | At least two clearly identified and documented relapses in the 2 years prior to entry, onset of the first relapse at least 1 year before randomisation | NR | NR | NR | NR | NR | NR |
| DEFINE^32^ | 320 and 794 | Dimethyl fumarate | RRMS | At least one clinically documented relapse within 12 months before randomization or a brain magnetic resonance imaging (MRI) scan, obtained within 6 weeks before randomization, that showed at least one gadolinium-enhancing lesion. | NR | NR | NR | NR | NR | NR |
| DELIVER^77^ | ID6369 | Natalizumab | RMS | Activity level not specified | NR | NR | NR | NR | NR | NR |
| ECGAS2001^41^ | 527 | Glatiramer acetate | RRMS | At least one documented relapse in the preceding 2 years, and at least one enhancing lesion on their screening brain MRI. | NR | NR | NR | NR | NR | NR |
| ESG 1998^42^ | 527 | INF-β 1b | SPMS | Two relapses or more or 1·0 point or more increase in EDSS in the previous 2 years. | NA | NA | NA | NA | NA | NA |
| Etemedifar 2006^43^ | 527 | INF-β 1a and 1b | RRMS | >=2relapses within the 2-year period to treatment initiation | NR | NR | NR | NR | NR | NR |
| EVIDENCE^44^ | 527 | INF-β 1a | RRMS | At least two exacerbations of MS in the prior 2 years. | NR | NR | NR | NR | NR | NR |
| EVOLVE-MS-2^81^ | 794 | Diroximel fumarate and Dimethyl fumarate | RRMS | Active not in scope | NR | NR | NR | NR | NR | NR |
| EXPAND^67^ | 656 | Siponimod | SPMS | at least one relapse in the previous 2 years | NA | NA | NA | NA | NA | NA |
| FREEDOMS I^21^ | 254 | Fingolimod | RRMS | One or more documented relapses in the previous year or two or more in the previous 2 years | Highly active | NR | Patients who had (1) ≥1 relapse in the previous year and either ≥1 gadolinium (Gd) enhancing T1 lesion or ≥9 T2 lesions at baseline and/or (2) as many or more relapses in the year before baseline as in the previous year despite previous DMT use | NR | NR | NR |
| FREEDOMS II^22^ | 254 | Fingolimod | RRMS | One or more documented relapses in the previous year or two or more in the previous 2 years | Highly active | NR | Patients who had (1) ≥1 relapse in the previous year and either ≥1 gadolinium (Gd) enhancing T1 lesion or ≥9 T2 lesions at baseline and/or (2) as many or more relapses in the year before baseline as in the previous year despite previous DMT use | NR | NR | NR |
| FTY720 D220^24^ | 254 | Fingolimod | RMS | Two or more documented relapses during the previous 2 years, one or more documented relapses in the year before enrolment, and one or more gadolinium-enhanced lesions detected on magnetic resonance imaging (MRI) at screening. | NR | NR | NR | NR | NR | NR |
| GALA^45^ | 527 | Glatiramer acetate | RRMS | At least 1 documented relapse in the 12 months before screening | NR | NR | NR | NR | NR | NR |
| GATE^46^ | 527 | Glatiramer acetate | RRMS | At least1 documented relapse in the previous year, and 1 to 15 gadolinium-enhancing lesions on T1-weighted images on screening brain MRI | NR | NR | NR | NR | NR | NR |
| HMR1726D/2001^28^ | 303 | Teriflunomide | RRMS | Two documented relapses in the previous 3 years, and one clinical relapse during the preceding year. | NR | NR | NR | NR | NR | NR |
| IFNB^47^ | 527 | INF-β 1b | RRMS | At least 2 acute exacerbations during the previous 2 years | NR | NR | NR | NR | NR | NR |
| IMPROVE^48^ | 527 | INF-β 1b | RRMS | ≥1 clinical event and ≥1 gadolinium [Gd]-enhancing MRI lesion within the 6 months before randomization | NR | NR | NR | NR | NR | NR |
| INCOMIN^49^ | 527 | INF-β 1a and 1b | RRMS | Two clinically documented relapses during the preceding 2 years | NR | NR | NR | NR | NR | NR |
| Kappos 2008^82^ | 320 | Dimethyl fumarate | RRMS | Either at least one relapse within 12 months of randomisation and a previous cranial MRI scan showing lesions consistent with multiple sclerosis, or GdE lesions on MRI scans done within 6 weeks of randomisation. | NR | NR | NR | NR | NR | NR |
| KAPPOS 2011^50^ | 527 and 533 | Ocrelizumab | RRMS | Two or more documented relapses within 3 years before screening, at least one of which occurred within the past year | NR | NR | NR | NR | NR | NR |
| Knobler 1993^51^ | 527 | INF-β 1b | RRMS | NR | NR | NR | NR | NR | NR | NR |
| Mokhber 2014^53^ | 527 | INF-β 1a and 1b | MS | NR | NR | NR | NR | NR | NR | NR |
| MS 201^1^ | 127 | Natalizumab | RMS | Two or more exacerbations in the past 18 months; | NR | NR | NR | NR | NR | NR |
| MS 231^1^ | 127 | Natalizumab | RMS | At least 2 MS exacerbations within the past 2 years | NR | NR | NR | NR | NR | NR |
| MSCRG 1996^52^ | 527 | interferon beta 1a | RRMS | At least 2 documented exacerbations in the prior 3 years | NR | NR | NR | NR | NR | NR |
| NASG 2004 | 527 | INF-β 1b | SPMS | At least one relapse followed by progressive deterioration sustained for at least 6 months | NA | NA | NA | NA | NA | NA |
| NOVA^78^ | ID6369 | Natalizumab | RRMS | The study’s population does not include active RRMS | NR | NR | NR | NR | NR | NR |
| ONWARD^66^ | 616 and 1053 | Cladribine and INF-β 1a | RMS | The study’s population does not include active RRMS | Active relapsing disease despite interferon beta treatment | NR | ≥ 1 MS relapse during treatment with Interferon beta for ≥48 consecutive weeks before screening | NR | NR | NR |
| OPERA I^62^ | 533 | Ocrelizumab | RMS | At least two documented clinical re-lapses within the previous 2 years or one clinical relapse within the year before screening | Highly active | NR | Patients treated with interferons or glatiramer acetate for at least 1 year, and: Had at least one relapse in the previous year, and Had at least one T1 Gd-enhancing lesion on brain MRI at baseline, or Had at least nine T2 hyperintense lesions on brain MRI at baseline (post hoc analysis) | RES | Patients had at least two relapses in the previous year, and Had at least one T1 Gd-enhancing lesion on brain MRI at baseline, or had an increase in T2 hyperintense lesion count on brain MRI at baseline (changing from 0-5 to 6-9, >9 lesions or 6-9 lesions to >9 lesions), compared to previous MRI | No |
| OPERA II^62^ | 533 | Ocrelizumab | RMS | At least two documented clinical re-lapses within the previous 2 years or one clinical relapse within the year before screening | Highly active | NR | Patients treated with interferons or glatiramer acetate for at least 1 year, and: Had at least one relapse in the previous year, and Had at least one T1 Gd-enhancing lesion on brain MRI at baseline, or Had at least nine T2 hyperintense lesions on brain MRI at baseline (post hoc analysis) | RES | Patients had at least two relapses in the previous year, and Had at least one T1 Gd-enhancing lesion on brain MRI at baseline, or had an increase in T2 hyperintense lesion count on brain MRI at baseline (changing from 0-5 to 6-9, >9 lesions or 6-9 lesions to >9 lesions), compared to previous MRI | No |
| OPTIMUM^73^ | 767 | Ponesimod and teriflunomide | RMS | One or more MS attacks with onset within the period of 12 to 1 months prior to randomization, or by two or more MS attacks with onset within the 24 to 1 months prior to randomization, or with one or more gadolinium-enhancing (Gd+) lesion(s) of the brain on an MRI performed within 6 months prior to randomization. | Highly active | NR | ≥2 relapses within the 1 year prior to study entry and baseline EDSS score >2 and baseline MRI ≥1 Gd+ T1 lesion or any DMT received within 12 months prior to randomization and one or both of the following: 1) ≥1 relapse within 1 year prior to study entry and the baseline MRI either ≥1 Gd+ T1 lesion and/or ≥9 T2 lesions or 2) number of relapses within 1 year prior to study entry ≥ number of relapses between 2 and 1 year prior to study entry, for patients with ≥1 relapse within 2 years prior to study entry. | Not specified | NR | Yes |
| ORATORIO | 585 | Ocrelizumab | PPMS | Patients with early PPMS with evidence of gadolinium-enhancing T1 lesions at screening or baseline or new T2 lesions between screening and baseline | NA | NA | NA | NA | NA | NA |
| OWIMS^55^ | 527 | INF-β 1a | RRMS | Patients had experienced at least one relapse in the prior 24 months but not in the 8 weeks before entry. At least three lesions consistent with MS were required on a screening MRI. | NR | NR | NR | NR | NR | NR |
| PRISMS^56^ | 527 | INF-β 1a | RRMS | At least two relapses in the preceding 2 years | NR | NR | NR | NR | NR | NR |
| RADIANCE part A^69^ | 706 | Ozanimod and INF-β 1a | RMS | At least one relapse within 12 months before screening or at least one relapse within 24 months before screening plus at least one gadolinium-enhancing lesion within the 12 months | NR | NR | NR | NR | NR | NR |
| RADIANCE part B^70^ | 706 | Ozanimod and INF-β 1a | RMS | At least one relapse within 12 months before screening or at least one relapse within 24 months before screening plus at least one gadolinium-enhancing lesion within the 12 months | NR | NR | NR | NR | NR | NR |
| REFINE^79^ | ID6369 | Natalizumab | RRMS | Active not in scope | NR | NR | NR | NR | NR | NR |
| REFORMS^57^ | 527 | INF-β 1a and 1b | RRMS | NR | NR | NR | NR | NR | NR | NR |
| REGARD^58^ | 527 | INF-β 1a | RRMS | At least one attack in the preceding 12 months | NR | NR | NR | NR | NR | NR |
| REMAIN^59^ | 527 | INF-β 1a | RMS | Active not in scope | NR | NR | NR | NR | NR | NR |
| RPC01-1001^71^ | 706 | Ozanimod | RMS | Activity level not specified | NR | NR | NR | NR | NR | NR |
| SENTINEL^20^ | 127 | Natalizumab | RRMS | At least one relapse during the 12-month period before randomization | Highly active | NR | ≥ 2 relapses in the year before study entry and ≥ 1 Gd+ lesion at study entry). | NR | NR | NR |
| SPECTRIMS^60^ | 527 | INF-β 1a | SPMS | 1 relapse during the previous 2 years | NA | NA | NA | NA | NA | NA |
| SUNBEAM^72^ | 706 | Ozanimod and INF-β 1a | RMS | At least one relapse in the 12 months before screening or at least one relapse in the 24 months before screening plus at least one gadolinium-enhancing lesion in the 12 months before randomisation. | NR | NR | NR | NR | NR | NR |
| TEMSO^25^ | 303 | Teriflunomide | RMS | At least two clinical relapses in the previous 2 years or one relapse during the preceding year | NR | NR | NR | NR | NR | NR |
| TENERE^27^ | 303 | Teriflunomide | RMS | Activity level not specified | NR | NR | NR | NR | NR | NR |
| TOWER^26^ | 303 | Teriflunomide | RMS | At least one relapse in the previous year or at least two relapses in the previous 2 years | NR | NR | NR | NR | NR | NR |
| TRANSFORMS^21^ | 254 | Fingolimod | RRMS | At least one documented relapse during the previous year or at least two documented relapses during the previous 2 year | Highly active | High disease activity despite previous DMT | Patients who received any DMT during the year before study enrolment who had as many or more relapses in the year before study entry than in the year 2 years before study entry, patients who received any DMT during the year before study enrolment and had ≥1 relapse in the previous year plus≥1 Gd-enhancing T1 lesion or ≥9 T2 lesions at baseline, and treatment naïve patients with rapidly evolving severe RRMS, defined as ≥2 relapses within the year before baseline and ≥1 Gd-enhancing T1 lesion at baseline. | RES | ≥2 relapses within the year before baseline and ≥1 Gd-enhancing T1 lesion at baseline. | Yes |
| ULTIMATE I^75^ | 1025 | Ublituximab and teriflunomide | RMS | At least two relapses in the previous 2 years, or one relapse or at least one gadolinium-enhancing lesion or both in the year before screening | Highly active | NR | ≥2 relapses in the year prior and ≥1 Gd+ T1 lesion at baseline. | Highly active | NR | Yes |
| ULTIMATE II^75^ | 1025 | Ublituximab and teriflunomide | RMS | At least two relapses in the previous 2 years, or one relapse or at least one gadolinium-enhancing lesion or both in the year before screening | Highly active | NR | ≥2 relapses in the year prior and ≥1 Gd+ T1 lesion at baseline. | Highly active | NR | Yes |

## References

1. National Institute for Health Care Excellence (NICE). [TA127] Natalizumab for the treatment of adults with highly active relapsing–remitting multiple sclerosis 2007 [Available from: <https://www.nice.org.uk/guidance/ta127>.

2. National Institute for Health Care Excellence (NICE). [TA254] Fingolimod for the treatment of highly active relapsing–remitting multiple sclerosis 2012 [Available from: <https://www.nice.org.uk/guidance/ta254>.

3. National Institute for Health Care Excellence (NICE). [TA303] Teriflunomide for treating relapsing–remitting multiple sclerosis 2014 [Available from: <https://www.nice.org.uk/guidance/ta303>.

4. National Institute for Health Care Excellence (NICE). [TA312] Alemtuzumab for treating highly active relapsing–remitting multiple sclerosis 2014 [Available from: <https://www.nice.org.uk/guidance/ta312>.

5. National Institute for Health Care Excellence (NICE). [TA320] Dimethyl fumarate for treating relapsing‑remitting multiple sclerosis 2014 [Available from: <https://www.nice.org.uk/guidance/ta320>.

6. National Institute for Health Care Excellence (NICE). [TA527] Beta interferons and glatiramer acetate for treating multiple sclerosis 2018 [Available from: <https://www.nice.org.uk/guidance/ta527>.

7. National Institute for Health Care Excellence (NICE). [TA533] Ocrelizumab for treating relapsing–remitting multiple sclerosis 2018 [Available from: <https://www.nice.org.uk/guidance/ta533>.

8. National Institute for Health Care Excellence (NICE). [TA585] Ocrelizumab for treating primary progressive multiple sclerosis 2019 [Available from: <https://www.nice.org.uk/guidance/ta585>.

9. National Institute for Health Care Excellence (NICE). [TA616] Cladribine for treating relapsing–remitting multiple sclerosis 2019 [Available from: <https://www.nice.org.uk/guidance/ta616>.

10. National Institute for Health Care Excellence (NICE). [TA656] Siponimod for treating secondary progressive multiple sclerosis 2020 [Available from: <https://www.nice.org.uk/guidance/ta656>.

11. National Institute for Health Care Excellence (NICE). [TA624] Peginterferon beta-1a for treating relapsing–remitting multiple sclerosis 2020 [Available from: <https://www.nice.org.uk/guidance/ta624>.

12. National Institute for Health Care Excellence (NICE). [TA699] Ofatumumab for treating relapsing multiple sclerosis 2021 [Available from: <https://www.nice.org.uk/guidance/ta699>.

13. National Institute for Health Care Excellence (NICE). [TA706] Ozanimod for treating relapsing–remitting multiple sclerosis 2021 [Available from: <https://www.nice.org.uk/guidance/ta706>.

14. National Institute for Health Care Excellence (NICE). [TA767] Ponesimod for treating relapsing–remitting multiple sclerosis 2022 [Available from: <https://www.nice.org.uk/guidance/ta767>.

15. National Institute for Health Care Excellence (NICE). [TA794] Diroximel fumarate for treating relapsing–remitting multiple sclerosis 2022 [Available from: <https://www.nice.org.uk/guidance/ta794>.

16. National Institute for Health Care Excellence (NICE). [TA1025] Ublituximab for treating relapsing multiple sclerosis: NICE; 2025 [Available from: <https://www.nice.org.uk/guidance/ta1025>.

17. National Institute for Health Care Excellence (NICE). [TA 1053] Cladribine for treating active relapsing forms of multiple sclerosis. 2025.

18. National Institute for Health Care E. [TA ID6369] Natalizumab (originator and biosimilar) for treating highly active relapsing–remitting multiple sclerosis after disease-modifying therapy. 2025.

19. Polman CH, O'Connor PW, Havrdova E, Hutchinson M, Kappos L, Miller DH, et al. A Randomized, Placebo-Controlled Trial of Natalizumab for Relapsing Multiple Sclerosis. The New England Journal of Medicine. 2006;354(9):899-910.

20. Rudick RA, Stuart WH, Calabresi PA, Confavreux C, Galetta SL, Radue E-W, et al. Natalizumab plus interferon beta-1a for relapsing multiple sclerosis. The New England journal of medicine. 2006;354(9):911-23.

21. Kappos L, Radue E-W, O'Connor P, Polman C, Hohlfeld R, Calabresi P, et al. A placebo-controlled trial of oral fingolimod in relapsing multiple sclerosis. The New England Journal of Medicine. 2010;362(5):387-401.

22. Calabresi PA, Radue E-W, Goodin D, Jeffery D, Rammohan KW, Reder AT, et al. Safety and efficacy of fingolimod in patients with relapsing-remitting multiple sclerosis (FREEDOMS II): a double-blind, randomised, placebo-controlled, phase 3 trial. The Lancet Neurology. 2014;13(6):545-56.

23. Barkhof F, de Jong R, Sfikas N, de Vera A, Francis G, Cohen J. The influence of patient demographics, disease characteristics and treatment on brain volume loss in Trial Assessing Injectable Interferon vs FTY720 Oral in Relapsing-Remitting Multiple Sclerosis (TRANSFORMS), a phase 3 study of fingolimod in multiple sclerosis. Multiple Sclerosis 2014;20(13):1704-13.

24. Kappos L, Antel J, Comi G, Montalban X, O'Connor P, Polman CH, et al. Oral fingolimod (FTY720) for relapsing multiple sclerosis. The New England journal of medicine. 2006;355(11):1124-40.

25. O'Connor P, Wolinsky JS, Confavreux C, Comi G, Kappos L, Olsson TP, et al. Randomized trial of oral teriflunomide for relapsing multiple sclerosis. N Engl J Med. 2011;365(14):1293-303.

26. Confavreux C, O'Connor P, Comi G, Freedman MS, Miller AE, Olsson TP, et al. Oral teriflunomide for patients with relapsing multiple sclerosis (TOWER): a randomised, double-blind, placebo-controlled, phase 3 trial. Lancet Neurology. 2014;13(3).

27. Vermersch P, Czlonkowska A, Grimaldi LM, Confavreux C, Comi G, Kappos L, et al. Teriflunomide versus subcutaneous interferon beta-1a in patients with relapsing multiple sclerosis: a randomised, controlled phase 3 trial. Mult Scler. 2014;20(6):705-16.

28. O'Connor PW, Li D, Freedman MS, Bar-Or A, Rice GP, Confavreux C, et al. A Phase II study of the safety and efficacy of teriflunomide in multiple sclerosis with relapses. Neurology. 2006;66(6):894-900.

29. Coles AJ, Compston DAS, Selmaj KW, Lake SL, Moran S, Margolin DH, et al. Alemtuzumab vs. interferon beta-1a in early multiple sclerosis. The New England Journal of Medicine. 2008;359(17):1786-801.

30. Cohen JA, Coles AJ, Arnold DL, Confavreux C, Fox EJ, Hartung H-P, et al. Alemtuzumab versus interferon beta 1a as first-line treatment for patients with relapsing-remitting multiple sclerosis: a randomised controlled phase 3 trial. Lancet 2012;380(9856):1819-28.

31. Coles AJ, Twyman CL, Arnold DL, Cohen JA, Confavreux C, Fox EJ, et al. Alemtuzumab for patients with relapsing multiple sclerosis after disease-modifying therapy: a randomised controlled phase 3 trial. Lancet 2012;380(9856):1829-39.

32. Gold R, Kappos L, Arnold DL, Bar-Or A, Giovannoni G, Selmaj K, et al. Placebo-controlled phase 3 study of oral BG-12 for relapsing multiple sclerosis. N Engl J Med. 2012;367(12):1098-107.

33. Fox RJ, Miller DH, Phillips JT, Hutchinson M, Havrdova E, Kita M, et al. Placebo-controlled phase 3 study of oral BG-12 or glatiramer in multiple sclerosis. The New England Journal of Medicine. 2012;367(12):1087-97.

34. Kent S, Becker F, Feenstra T, Tran-Duy A, Schlackow I, Tew M, et al. The Challenge of Transparency and Validation in Health Economic Decision Modelling: A View from Mount Hood. Pharmacoeconomics. 2019;37(11):1305-12.

35. Cadavid D, Wolansky LJ, Skurnick J, Lincoln J, Cheriyan J, Szczepanowski K, et al. Efficacy of treatment of MS with IFNbeta-1b or glatiramer acetate by monthly brain MRI in the BECOME study. Neurology. 2009;72(23):1976-83.

36. O'Connor P, Filippi M, Arnason B, Comi G, Cook S, Goodin D, et al. 250 microg or 500 microg interferon beta-1b versus 20 mg glatiramer acetate in relapsing-remitting multiple sclerosis: a prospective, randomised, multicentre study. The Lancet Neurology. 2009;8(10):889-97.

37. Bornstein MB, Miller A, Slagle S. A pilot trial of cop 1 in exacerbating-remitting multiple sclerosis. New England Journal of Medicine. 1987;317(7):408-14.

38. Vollmer TL, Sorensen PS, Selmaj K, Zipp F, Havrdova E, Cohen JA, et al. A randomized placebo-controlled phase III trial of oral laquinimod for multiple sclerosis. Journal of neurology. 2014;261(4):773-83.

39. Calabrese M, Bernardi V, Atzori M, Mattisi I, Favaretto A, Rinaldi F, et al. Effect of disease-modifying drugs on cortical lesions and atrophy in relapsing-remitting multiple sclerosis. Multiple Sclerosis 2012;18(4):418-24.

40. Johnson KP, Brooks BR, Cohen JA, Ford CC, Goldstein J, Lisak RP, et al. Copolymer 1 reduces relapse rate and improves disability in relapsing-remitting multiple sclerosis: results of a phase III multicenter, double-blind placebo-controlled trial. The Copolymer 1 Multiple Sclerosis Study Group. Neurology. 1995;45(7):1268-76.

41. You S, Wan MP. Modeling and experiments of the adhesion force distribution between particles and a surface. Langmuir. 2014;30(23).

42. Placebo-controlled multicentre randomised trial of interferon beta-1b in treatment of secondary progressive multiple sclerosis. European Study Group on interferon beta-1b in secondary progressive MS. Lancet. 1998;352(9139):1491-7.

43. Etemadifar M, Janghorbani M, Shaygannejad V. Comparison of Betaferon, Avonex, and Rebif in treatment of relapsing-remitting multiple sclerosis. Acta Neurologica Scandinavica. 2006;113(5):283-7.

44. Panitch H, Goodin DS, Francis G, Chang P, Coyle PK, O'Connor P, et al. Randomized, comparative study of interferon beta-1a treatment regimens in MS: The EVIDENCE Trial. Neurology. 2002;59(10):1496-506.

45. Khan O, Rieckmann P, Boyko A, Selmaj K, Zivadinov R. Three times weekly glatiramer acetate in relapsing-remitting multiple sclerosis. Annals of Neurology. 2013;73(6):705-13.

46. Najafi B, Ghaderi H, Jafari M, Najafi S, Ahmad Kiadaliri A. Cost effectiveness analysis of Avonex and CinnoVex in Relapsing Remitting MS. Global Journal of Health Science. 2014;7(2).

47. Duquette P, Girard M, Despault L, DuBois R, Knobler RL, Lublin FD, et al. Interferon beta-1b is effective in relapsing-remitting multiple sclerosis. I. Clinical results of a multicenter, randomized, double-blind, placebo- controlled trial. Neurology. 1993;43(4 I):655-61.

48. De Stefano N, Sormani MP, Stubinski B, Blevins G, Drulovic JS, Issard D, et al. Efficacy and safety of subcutaneous interferon beta-1a in relapsing-remitting multiple sclerosis: further outcomes from the IMPROVE study. Journal of the Neurological Sciences. 2012;312(1-2):97-101.

49. Durelli L, Verdun E, Barbero P, Bergui M, Versino E, Ghezzi A, et al. Every-other-day interferon beta-1b versus once-weekly interferon beta-1a for multiple sclerosis: results of a 2-year prospective randomised multicentre study (INCOMIN). Lancet 2002;359(9316):1453-60.

50. Kappos L, Li D, Calabresi PA, O'Connor P, Bar-Or A, Barkhof F, et al. Ocrelizumab in relapsing-remitting multiple sclerosis: a phase 2, randomised, placebo-controlled, multicentre trial. Lancet 2011;378(9805):1779-87.

51. Knobler RL, Greenstein JI, Johnson KP, Lublin FD, Panitch HS, Conway K, et al. Systemic recombinant human interferon-beta treatment of relapsing-remitting multiple sclerosis: pilot study analysis and six-year follow-up. Journal of interferon research. 1993;13(5):333-40.

52. Jacobs LD, Cookfair DL, Rudick RA, Herndon RM, Richert JR, Salazar AM, et al. Intramuscular interferon beta-1a for disease progression in relapsing multiple sclerosis. The Multiple Sclerosis Collaborative Research Group (MSCRG). Annals of Neurology. 1996;39(3):285-94.

53. Mokhber N, Azarpazhooh A, Orouji E, Rao SM, Khorram B, Sahraian MA, et al. Cognitive dysfunction in patients with multiple sclerosis treated with different types of interferon beta: a randomized clinical trial. J Neurol Sci. 2014;342(1-2):16-20.

54. Panitch H, Miller A, Paty D, Weinshenker B. Interferon beta-1b in secondary progressive MS: results from a 3-year controlled study. Neurology. 2004;63(10):1788-95.

55. Wei H, Yan F, Chen X, Zhang H, Cheng Q, Xue D, et al. Large-aperture space optical system testing based on the scanning Hartmann. Applied Optics. 2017;56(8).

56. Ebers GC, Rice G, Lesaux J, Paty D, Oger J, Li DKB, et al. Randomised double-blind placebo-controlled study of interferon beta-1a in relapsing/remitting multiple sclerosis. Lancet. 1998;352(9139):1498-504.

57. Singer B, Bandari D, Cascione M, LaGanke C, Huddlestone J, Bennett R, et al. Comparative injection-site pain and tolerability of subcutaneous serum-free formulation of interferonbeta-1a versus subcutaneous interferonbeta-1b: results of the randomized, multicenter, Phase IIIb REFORMS study. BMC neurology. 2012;12:154.

58. Mikol DD, Barkhof F, Chang P, Coyle PK, Jeffery DR, Schwid SR, et al. Comparison of subcutaneous interferon beta-1a with glatiramer acetate in patients with relapsing multiple sclerosis (the REbif vs Glatiramer Acetate in Relapsing MS Disease [REGARD] study): a multicentre, randomised, parallel, open-label trial. The Lancet Neurology. 2008;7(10):903-14.

59. Rieckmann P, Heidenreich F, Sailer M, Zettl UK, Zessack N, Hartung H-P, et al. Treatment de-escalation after mitoxantrone therapy: results of a phase IV, multicentre, open-label, randomized study of subcutaneous interferon beta-1a in patients with relapsing multiple sclerosis. Therapeutic advances in neurological disorders. 2012;5(1):3-12.

60. Anonymous. Randomized controlled trial of interferon- beta-1a in secondary progressive MS: Clinical results. Neurology. 2001;56(11):1496-504.

61. Boyko OV, Khoroshylova II, Petrov SV, Lush NY, Guseva ME, Boyko AN. [Additional possible mechanisms of the action of ocrelizumab in multiple sclerosis on example of a case-report]. Vozmozhnye dopolnitel'nye mekhanizmy deistviia okrelizumaba pri rasseiannom skleroze na primere klinicheskogo sluchaia. 2018;118(8. Vyp. 2):116-20.

62. Hauser SL, Bar-Or A, Comi G, Giovannoni G, Hartung H-P, Hemmer B, et al. Ocrelizumab versus Interferon Beta-1a in Relapsing Multiple Sclerosis. The New England Journal of Medicine. 2017;376(3):221-34.

63. Masterman D, Fontoura P, Belachew S, Garren H, Mairon N, Chin P, et al. Ocrelizumab versus Placebo in Primary Progressive Multiple Sclerosis. The New England journal of medicine. 2017;376(3).

64. Giovannoni G, Comi G, Cook S, Rammohan K, Rieckmann P, Soelberg Sorensen P, et al. A placebo-controlled trial of oral cladribine for relapsing multiple sclerosis. The New England Journal of Medicine. 2010;362(5):416-26.

65. Leist TP, Comi G, Cree BAC, Coyle PK, Freedman MS, Hartung H-P, et al. Effect of oral cladribine on time to conversion to clinically definite multiple sclerosis in patients with a first demyelinating event (ORACLE MS): a phase 3 randomised trial. The Lancet Neurology. 2014;13(3):257-67.

66. Montalban X, Leist TP, Cohen BA, Moses H, Campbell J, Hicking C, et al. Cladribine tablets added to IFN-beta in active relapsing MS. Neurology: Neuroimmunology and NeuroInflammation. 2018;5(5).

67. Kappos L, Bar-Or A, Cree BAC, Fox RJ, Giovannoni G, Gold R, et al. Siponimod versus placebo in secondary progressive multiple sclerosis (EXPAND): a double-blind, randomised, phase 3 study. The Lancet. 2018;391(10127).

68. Hauser SL, Bar-Or A, Cohen JA, Comi G, Correale J, Coyle PK, et al. Ofatumumab versus Teriflunomide in Multiple Sclerosis. The New England Journal of Medicine. 2020;383(6):546-57.

69. Cohen JA, Arnold DL, Comi G, Bar-Or A, Gujrathi S, Hartung JP, et al. Safety and efficacy of the selective sphingosine 1-phosphate receptor modulator ozanimod in relapsing multiple sclerosis (RADIANCE): a randomised, placebo-controlled, phase 2 trial. Lancet Neurology. 2016;15(4).

70. Cohen JA, Comi G, Selmaj KW, Bar-Or A, Arnold DL, Steinman L, et al. Safety and efficacy of ozanimod versus interferon beta-1a in relapsing multiple sclerosis (RADIANCE): a multicentre, randomised, 24-month, phase 3 trial. The Lancet Neurology. 2019;18(11):1021-33.

71. Harris S, Tran JQ, Southworth H, Spencer CM, Cree BAC, Zamvil SS. Effect of the sphingosine-1-phosphate receptor modulator ozanimod on leukocyte subtypes in relapsing MS. Neurol Neuroimmunol Neuroinflamm. 2020;7(5).

72. Comi G, Kappos L, Selmaj KW, Bar-Or A, Arnold DL, Steinman L, et al. Safety and efficacy of ozanimod versus interferon beta-1a in relapsing multiple sclerosis (SUNBEAM): a multicentre, randomised, minimum 12-month, phase 3 trial. The Lancet Neurology. 2019;18(11):1009-20.

73. Kappos L, Fox RJ, Burcklen M, Freedman MS, Havrdova EK, Hennessy B, et al. Ponesimod Compared With Teriflunomide in Patients With Relapsing Multiple Sclerosis in the Active-Comparator Phase 3 OPTIMUM Study: A Randomized Clinical Trial. JAMA Neurology. 2021;78(5):558-67.

74. Olsson T, Boster A, Fernandez O, Freedman MS, Pozzilli C, Bach D, et al. Oral ponesimod in relapsing-remitting multiple sclerosis: a randomised phase II trial. Journal of Neurology, Neurosurgery, and Psychiatry. 2014;85(11):1198-208.

75. Steinman L, Fox E, Hartung HP, Alvarez E, Qian P, Wray S, et al. Ublituximab versus Teriflunomide in Relapsing Multiple Sclerosis. N Engl J Med. 2022;387(8):704-14.

76. Hemmer B, Wiendl H, Roth K, Wessels H, Hofler J, Hornuss C, et al. Efficacy and Safety of Proposed Biosimilar Natalizumab (PB006) in Patients With Relapsing-Remitting Multiple Sclerosis: The Antelope Phase 3 Randomized Clinical Trial. JAMA Neurology. 2023;80(3):298-307.

77. Plavina T, Fox EJ, Lucas N, Muralidharan KK, Mikol D. A Randomized Trial Evaluating Various Administration Routes of Natalizumab in Multiple Sclerosis. J Clin Pharmacol. 2016;56(10):1254-62.

78. Peyrin-Biroulet L, Christopher R, Behan D, Lassen C. Modulation of sphingosine-1-phosphate in inflammatory bowel disease. Autoimmunity Reviews. 2017;16(5).

79. Trojano M, Ramio-Torrenta L, Grimaldi LM, Lubetzki C, Schippling S, Evans KC, et al. A randomized study of natalizumab dosing regimens for relapsing-remitting multiple sclerosis. Multiple sclerosis (Houndmills, Basingstoke, England). 2021;27(14):2240-53.

80. Lublin FD, Cofield SS, Cutter GR, Conwit R, Narayana PA, Nelson F, et al. Randomized study combining interferon and glatiramer acetate in multiple sclerosis. Annals of Neurology. 2013;73(3):327-40.

81. Naismith RT, Wundes A, Ziemssen T, Jasinska E, Freedman MS, Lembo AJ, et al. Diroximel Fumarate Demonstrates an Improved Gastrointestinal Tolerability Profile Compared with Dimethyl Fumarate in Patients with Relapsing-Remitting Multiple Sclerosis: Results from the Randomized, Double-Blind, Phase III EVOLVE-MS-2 Study. CNS Drugs. 2020;34(2):185-96.

82. Kappos L, Gold R, Miller DH, Macmanus DG, Havrdova E, Limmroth V, et al. Efficacy and safety of oral fumarate in patients with relapsing-remitting multiple sclerosis: a multicentre, randomised, double-blind, placebo-controlled phase IIb study. Lancet. 2008;372(9648):1463-72.
